# An in vivo model for overloading-induced soft tissue injury

**DOI:** 10.1038/s41598-022-10011-7

**Published:** 2022-04-11

**Authors:** Panagiotis E. Chatzistergos, Nachiappan Chockalingam

**Affiliations:** Centre for Biomechanics and Rehabilitation Technologies, School of Life Sciences and Education, Staffordshire University, Leek Road, Stoke-on-Trent, ST4 2DF UK

**Keywords:** Health care, Medical research, Engineering, Materials science

## Abstract

This proof-of-concept study demonstrates that repetitive loading to the pain threshold can safely recreate overloading-induced soft tissue damage and that localised tissue stiffening can be a potential marker for injury. This concept was demonstrated here for the soft tissue of the sole of the foot where it was found that repeated loading to the pain threshold led to long-lasting statistically significant stiffening in the overloaded areas. Loading at lower magnitudes did not have the same effect. This method can shed new light on the aetiology of overloading injury in the foot to improve the management of conditions such as diabetic foot ulceration and heel pain syndrome. Moreover, the link between overloading and tissue stiffening, which was demonstrated here for the first time for the plantar soft tissue, opens the way for an assessment of overloading thresholds that is not based on the subjective measurement of pain thresholds.

## Introduction

When a tissue is overloaded, its internal stresses exceed its capacity to carry load without damage (i.e., its strength) leading to injury. Our inability to quantitatively define overloading and assess in vivo tissue strength on a subject-specific basis remains a major barrier for the effective prevention of such injuries. To address this challenge, the present study proposes to use a person’s sensation of pain within in vivo mechanical testing to safely recreate and study overloading. As a first step, this concept will be demonstrated for the soft tissues of the sole of the foot (a.k.a. plantar soft tissue). Plantar soft tissue was selected because it is extremely sensitive to pain, it is accessible for imaging and it can be easily loaded (and overloaded) in a clinically relevant manner.

Another reason for selecting plantar soft tissue is that overlading in this tissue is also very important clinically^1^. If loading in the foot becomes too high, then pain “motivates” us to change the way we load the foot to offload overloaded regions. The protective role of pain against overloading becomes even clearer if one considers the implications of impaired pain sensation. For example, people with diabetes who lose the sensation of pain in their feet due to peripheral neuropathy, also tend to repeatedly overload and seriously injure their feet, leading even to amputation. In the UK alone, 169 people have a toe, foot or leg amputation every week due to diabetes^2^.

Based on these, the load that causes minimal pain in the healthy plantar soft tissue could be assumed to be a threshold for tissue overloading. If this is correct then, repetitive loading to the pain threshold should cause internal tissue damage. What is unclear is whether the extent of induced damage will be ethically acceptable and at the same time reliably detectable.

From an engineering perspective, tissue damage will also change the tissue’s mechanical behaviour^3^. In turn, this could also mean that the changes in tissue biomechanics that tissue damage has triggered could also be used to detect damage. Recent advances in non-invasive and clinically applicable methods to quantitatively assess the mechanical behaviour of soft tissues, such as clinical shear wave (SW) elastography, could significantly help to this end^4–8^.

The link between tissue damage and altered mechanical properties was previously demonstrated for muscle tissue using an animal model^9,10^. Nelissen et al. induced deep-tissue injury by applying large-strain indentation for two hours in the tibialis anterior muscle of anaesthetised rats^9^. Measurement of the tissue’s mechanical behaviour before and after overloading revealed significant localised stiffening following overload injury. Even though the methods for overloading-induced tissue damage presented by Nelissen et al. are not transferable to in vivo human testing, their findings highlight tissue stiffening as a potential marker for soft tissue overloading^9,10^.

Biological tissues are dynamic structures^6^ with a capacity for self-repair. Also, the mechanical behaviour of all soft tissues is strongly affected by their recent loading history. As a result, any loading will affect the tissue’s mechanical behaviour. Studies demonstrate that using a well-defined preconditioning protocol can minimise the effect of loading history to reveal underlying clinically relevant changes in tissue biomechanics^11,12^.

Based on the above, it can be hypothesised that if the pain threshold is indeed a threshold for overloading in the healthy feet, then repetitive loading of that magnitude should trigger localised changes in tissue stiffness^10^ that cannot be reversed by preconditioning. Repetitive loading of lower magnitude should not have the same effect. Careful selection of the number of overloading cycles should enable controlling the extent of the induced internal tissue damage to levels that can easily heal without further complications.

## Results

The aforementioned hypothesis was tested in the heels of 26 participants with healthy sensitivity in their feet (male/female: 16/10, age: 36 ± 10 years, BMI: 26.5 kg/m^2^ ± 6.5 kg/m^2^). All methods were carried out in accordance with relevant guidelines and regulations. Institutional ethical approval was obtained prior to the start of the study by Staffordshire University’s ethics committee and written informed consent was obtained from participants prior to initiation of study procedures.

Before testing, the left and right foot of each participant was randomly assigned as A or B and healthy sensitivity was confirmed using a neurothesiometer^13,14^. Tables of all collected data can be found in Supplementary Data S1.

**Figure 1:**
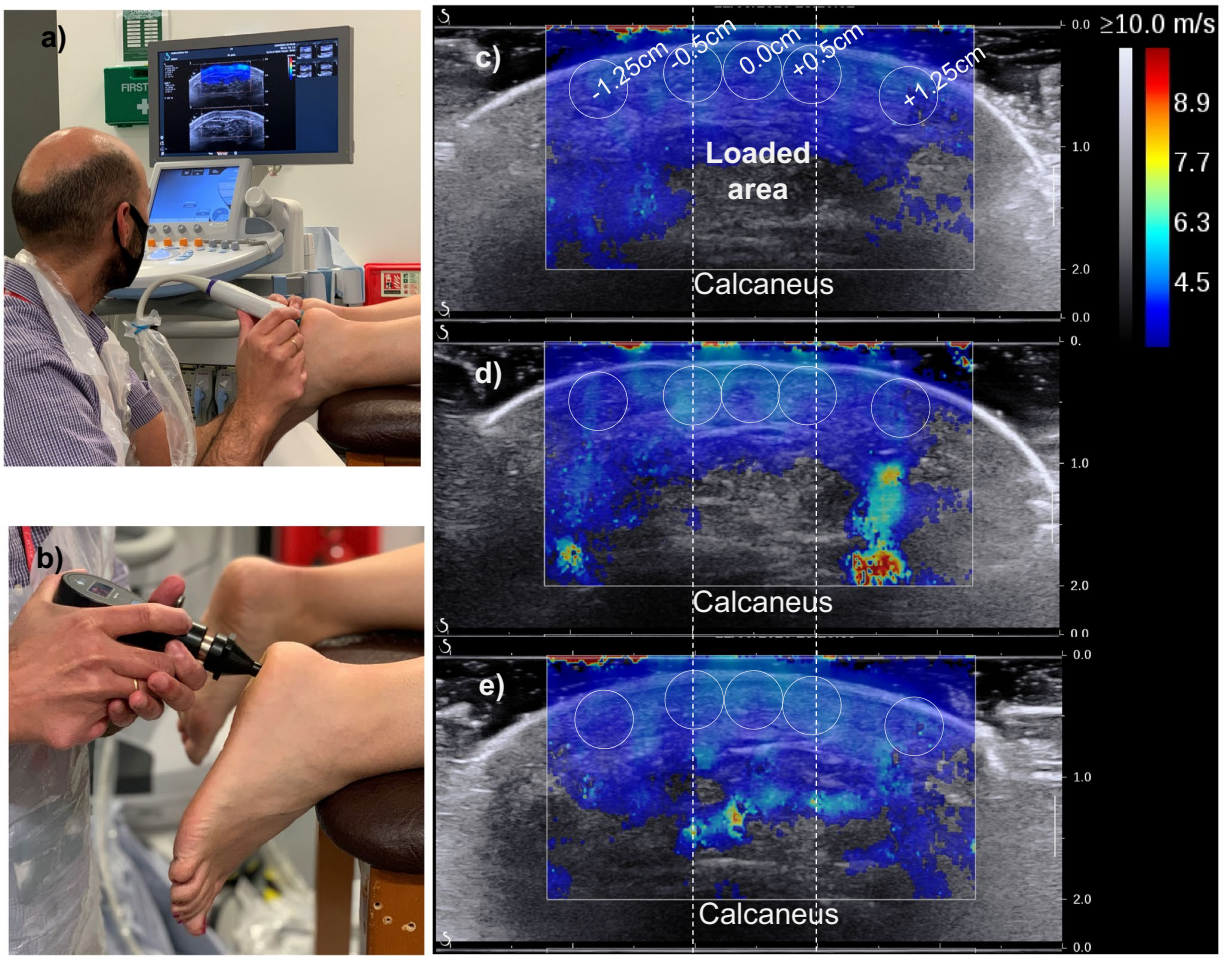
The testing set-up and an example of how SW elastography images were analysed. (**a**) The set-up for imaging and (**b**) loading the heel using a pressure algometer. SW elastograms at baseline (**c**), after overloading (**d**) or after walking/ preconditioning (**e**). The loaded area and circular areas for measuring SW speed at different distances from the centre are also shown.

Localised changes in plantar soft tissue stiffness were assessed by measuring changes in the distribution of SW propagation speed in an axial imaging plane at the centre of the heel (Fig. 1a). Previous research from the authors of this study had shown that increased or decreased SW speed in the plantar soft tissue is a reliable indicator for tissue stiffening or softening respectively^4^. To precondition the plantar soft tissue, the participants were asked to walk barefoot the length of the lab twice before imaging (≈ 80 steps). This preconditioning protocol was selected based on a preliminary test indicating that 80 steps of walking at self-selected speed could minimise the effect of loading history on SW speed in the heel. A detailed description of this test can be found in “Methods”.

**Figure 2:**
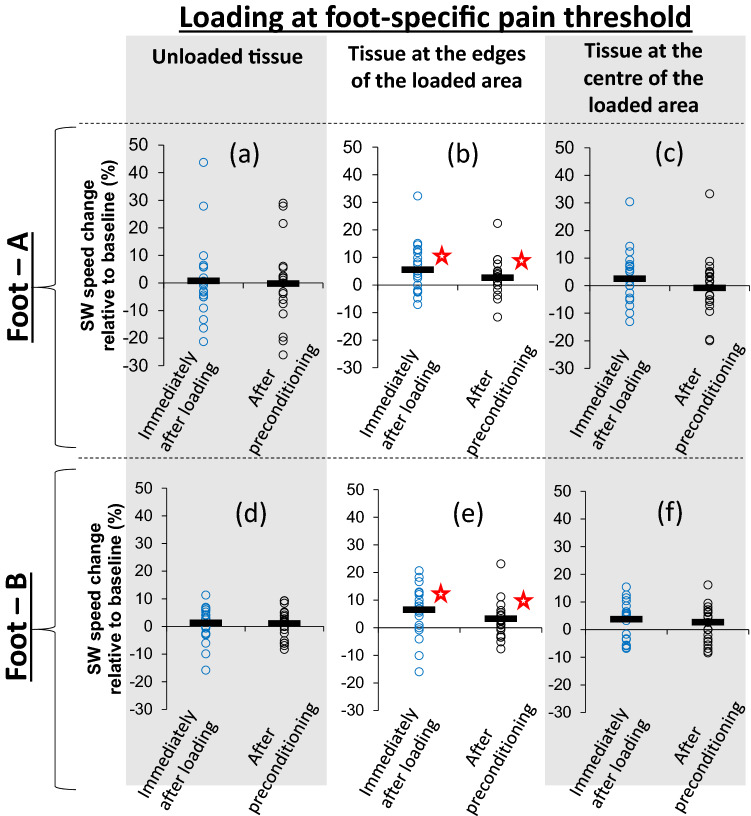
Scatterplots indicating whether loading at the foot-specific pain threshold led to localised stiffening and whether stiffening was eliminated by preconditioning. Open circles show measurements of change in SW speed relative to baseline for each participant and black lines show the average for each group of measurements^29^. Results are presented separately for foot-A (**a**–**c**) and foot-B (**d**–**f**) and for three different areas of the tissue, namely outside the loaded area (**a**,**d**), the edges of the loaded area (**b**,**e**) and the centre of the loaded area (**c**,**f**). Statistically significant changes in SW speed are indicated with “”.

Starting with foot-A, the heel was preconditioned before baseline imaging (Fig. 1c) and followed by ten cycles of loading to the participant’s pain threshold. A small area at the centre of the heel was loaded using a standardised handheld pressure algometer with a flat circular footprint (diameter = 1 cm) and the minimum force that triggered a sensation of mild pain as recorded (Fig. 1b). Imaging was repeated immediately after loading and after another round of preconditioning (Fig. 1d,e). Paired samples *t* test revealed statistically significant stiffening immediately after loading at the edges of the loaded area which was not eliminated by preconditioning (Fig. 2b). On average, SW speed increased by 5.6% relative to baseline immediately after repeated loading to the pain threshold (95% CI: − 0.497/− 0.111, t = − 3.256, df = 24, *P* = 0.003). Even though preconditioning reduced this difference, SW speed at the edges of the loaded area remained (on average) 2.7% higher than baseline, a statistically significant difference (95% CI: − 0.291/− 0.033, t = − 2.591, df = 24, *P* = 0.016). No significant change (*P* ≥ 0.05) was found at the centre of the loaded area (Fig. 2c) or outside the loaded area (Fig. 2a).

**Figure 3:**
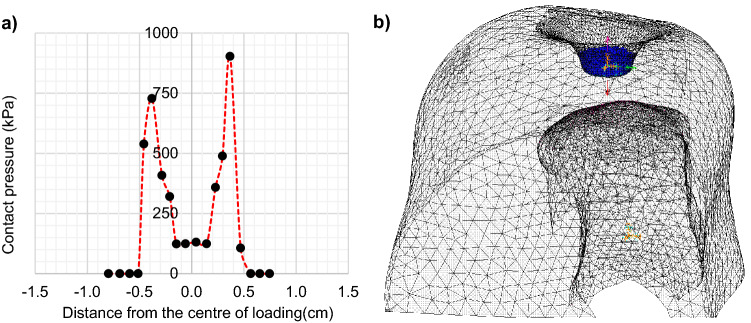
The numerically estimated distribution of contact pressure on the surface of the loaded area (**a**) and the finite element model used for these calculations (**b**). The FE model is presented in its deformed state for maximum compression.

Tissue stiffening was assessed separately for the edges of the loaded area since the pressure in those areas was expected to be the highest^15^. This was also confirmed for the specific loading scenario presented here using a previously validated finite element model^11^ of the heel (Fig. 3).

The same process of baseline imaging, imaging immediately after repetitive loading and after preconditioning was repeated three times for foot-B for increasing magnitudes of loading. Using the pain force thresholds (PFT) of foot-A (PFT-A) as a reference, loading was first applied to foot-B at 50%, then 80% of PFT-A before going to the foot-specific pain threshold (PFT-B) during the third and final round of loading and imaging. To eliminate any accumulated effects of loading, any change in SW speed in foot-B was assessed relative to measurements taken immediately before the start of each loading round.

Like foot-A, also in foot-B loading at the foot-specific pain threshold led to statistically significant tissue stiffening at the edges of the loaded area which was not eliminated by preconditioning (Fig. 2e). More specifically, SW speed immediately after loading at the foot-specific pain threshold and after preconditioning was on average 6.6% (95% CI: − 0.552/− 0.113, t = − 3.120, df = 25, *P* = 0.005) and 2.9% (95% CI: − 0.355/− 0.025, t = − 2.374, df = 25, *P* = 0.026) higher than baseline respectively. Both differences were statistically significant. No significant change (*P* ≥ 0.05) was found at the centre of the loaded area (Fig. 2f) or outside the loaded area (Fig. 2d).

**Figure 4:**
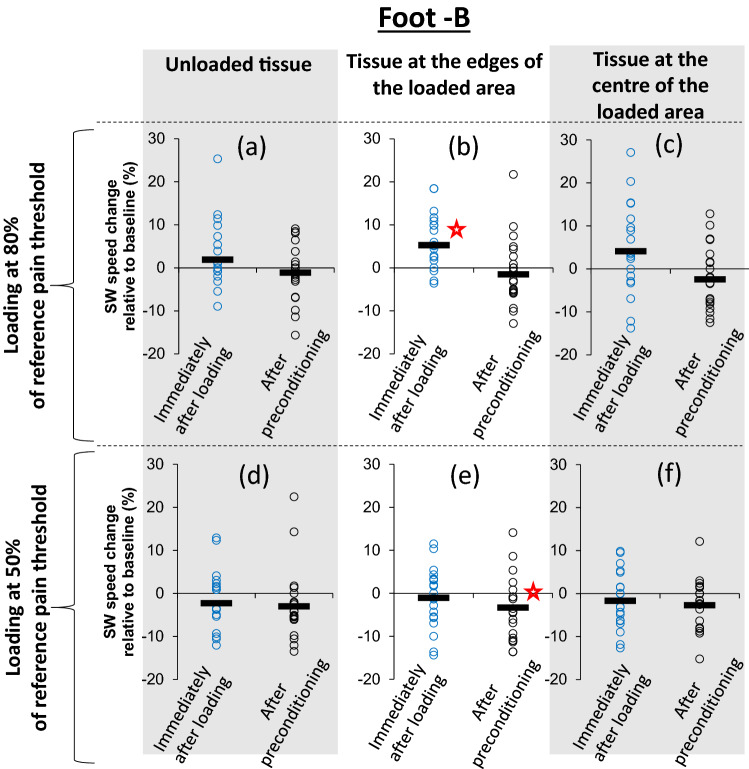
Scatterplots indicating whether loading in foot-B that was lower than the pain threshold caused significant changes in SW speed and whether these changes were eliminated by preconditioning. Open circles show measurements of change in SW speed relative to baseline for each participant and black lines show the average for each group of measurements^29^. Results are presented separately for loading equal to 80% (**a**–**c**) and 50% (**d**–**f**) of the reference pain threshold (PFT-A) and for three different areas of the tissue, namely outside the loaded area (**a**,**d**), the edges of the loaded area (**b**,**e**) and the centre of the loaded area (**c**,**f**). Statistically significant changes in SW speed are indicated with “”.

Loading at 80% of the reference pain threshold (PFT-A) led to statistically significant stiffening only at the edges of the loaded area immediately after loading (Fig. 4a–c). This time however stiffening was eliminated by preconditioning. As it can be seen in Fig. 4b, the average change in SW speed relative to baseline was equal to 5.3% (95% CI: − 0.440/− 0.087, t = − 3.087, df = 25, *P* = 0.005) immediately after loading, but dropped to practically zero and lost its statistical significance following preconditioning (− 1.5% difference to baseline, 95% CI: − 0.738/0.301, t = 1.248, df = 25, *P* = 0.224). No statistically significant stiffening was found for loading at 50% of the reference pain threshold. A consistent pattern of tissue softening was found instead (Fig. 4d–f). More specifically tissue softening was observed across all imaging regions with statistically significant softening at the edges of the loaded region following preconditioning (− 3.3% difference to baseline, 95% CI: 0.019/0.38, t = 2.281, df = 25, *P* = 0.031). A detailed list of all values shown in Figs. 2 and 4 can be found in Supplementary Data S2.

To get a sense of the longevity of the observed changes, a final round of imaging of foot-A was conducted immediately after testing and imaging for foot-B was completed. At that point ≈ 30 min had passed since foot-A was loaded to its pain threshold and it had been subjected to four additional preconditioning rounds (≈ 320 steps). Results indicated that stiffening at the edges of the loaded area persisted. More specifically the difference in SW speed relative to baseline was 3.6% and was statistically significant (95% CI: − 0.376/− 0.0244, t = − 2.350, df = 24, *P* = 0.027). The average difference to baseline for plantar soft tissue in the centre of the loaded area or outside the loaded area was 1.8% (95% CI: − 0.290/0.130, t = − 0.787, df = 24, *P* = 0.439) and 3.1% (95% CI: − 0.465/0.113, t = − 1.259, df = 24, *P* = 0.220) respective. Neither of these two differences was statistically significant.

At the end of the testing session, the participants were asked to walk the length of the lab one last time and to tell us whether there was any sensation of discomfort or pain in their heels. They were also instructed to contact us in case a sense of discomfort of pain started in the days following the test. None of the participants reported any issue while standing or walking at the end of the testing session or in the following days.

## Discussion

The results of this study confirm that repetitive loading to the threshold of pain triggers localised stiffening which is not eliminated by preconditioning and is consistent with the presence of tissue damage^9,10,16–18^. Localised stiffening was observed at the areas where pressure was most intense, namely at the edges of the loaded area (Fig. 2a). The fact that none of the participants reported any discomfort or pain after the end of the testing session or in the following days, verifies the safety of the proposed method.

In this study, changes in tissue biomechanics were assessed in 5 mm wide circular areas close to the skin surface (Fig. 1c–e). The effect of overloading in deeper tissues was not assessed because SW elastography cannot produce reliable measurements close to bony surfaces^4,5^. The use of alternative elastography techniques could enable the investigation of the effect of overloading on different plantar soft tissue layers^19^. More research will also be needed to test the applicability of the proposed methodology for shear overloading, which is another important contributor to overloading injuries^20^.

Another limitation of this study is that the duration of overloading-induced stiffening was not assessed. Previous research on the effect of overloading in the muscles of rats concluded that deep tissue injury leads to measurable localised stiffening in the affected tissue followed by softening (3–10 days post-injury) and a return to baseline stiffness 14 days post-injury^9^. The results presented here for the plantar soft tissue indicate that changes in plantar soft tissue biomechanics have sufficient longevity to enable their study in the lab. However, further research will be needed to explore the pattern and timescale of overloading-induced changes in plantar soft tissue.

Until now overloading thresholds for the foot have been assessed only for entire populations using retrospective epidemiological approaches^21–23^. Even though the measurement of overloading thresholds was beyond the scope of this proof-of-concept study, its methods and results can support further research in this direction. More specifically, the link between overloading, tissue damage and altered tissue biomechanics, which was demonstrated here for the first time for the plantar soft tissue, opens the way for an assessment of overloading thresholds that is not based on the subjective measurement of pain thresholds. Using the load that causes clinically relevant stiffening as a threshold for tissue overloading could enable the objective assessment of overloading thresholds and tissue strength in healthy and pathologic/insensitive tissues.

To this end, a reliable assessment of the interface loading between the load applicator and the plantar soft tissue would also be very important. In this study, overloading was imposed using a conventional pressure algometer (Fig.1b). Pressure algometers have flat load applicators and are commonly used to estimate pain pressure thresholds by dividing the PFT over the applicator’s area^15,24,25^. This calculation of pressure assumes that loading is evenly distributed across the surface of the applicator. However, previous literature^15^, and the numerical analysis included in this study, demonstrated that pressure at the edges of the pressure pain applicator is significantly higher compared to pressure at its centre (Fig. 2a). To account for our inability to accurately estimate pressure, pain thresholds were presented here in units of force.

The non-uniform distribution of pressure during loading could also explain the fact that statistically significant stiffening was observed only at the edges of the loaded area and not at its centre. In this case, the use of a different type of load applicator or indenter could lead to very different distribution of pressure and of localised stiffening. For example, the use of a hemispherical indenter would lead to high pressures developing at the centre of the loaded area, in which case significant localised stiffening would be expected at the centre and not at the edges of the loaded area. Further research will be needed to identify the most appropriate load applicator for the measurement of overloading thresholds and an accurate method to measure interface pressure.

In its current form, the presented methodology can be used to study how different areas of the foot are affected by pressure overloading and to shed new light on the aetiology of overload-induced injury. This can help improve the prevention and management of conditions which are triggered by overloading in the foot like DFU and heel pain syndrome^16,26^. At the same time, the presented concept can also open the way for the direct assessment of subject-specific thresholds for overloading in the plantar soft tissue, as well as in other soft tissues which, like the plantar soft tissue, are sensitive to pain, accessible for imaging and easily loaded in a clinically relevant manner (e.g., skin, muscle).

## Methods

### Participants

Adults with healthy sensitivity in their feet were included in this study. People with foot injuries or conditions which could affect their perception of pain were excluded. The specific inclusion/exclusion criteria used are as follows:

- Inclusion:
- Age ≥ 18 years AND
- healthy sensitivity in the feet.

- Exclusion criteria:
  Foot injury in the last 12 months OR
  presence of pain on the day of testing (anywhere in the body) OR
  diagnosis for a condition that could either cause pain or make the foot more sensitive to pain (e.g., gout, heel pain syndrome) OR
  diagnosis for a disorder that could affect pain perception (e.g., peripheral neuropathy).

All methods were carried out in accordance with relevant guidelines and regulations. Institutional ethical approval was obtained prior to the start of the study by Staffordshire University’s ethics committee and written informed consent was obtained from participants prior to data collection. Informed consent was obtained to publish the collected information and images in an online open-access publication.

### Confirmation of healthy sensitivity

A neurothesiometer (Horwell Scientific Laboratory Supplies) was used to measure vibration perception threshold (VPT) in each heel. VPT was measured at the centre of each heel three times and their average was used as the final VPT score^13,14^. Healthy sensitivity in the heels was defined as having VPT < 15 V in both heels^13^. The average (± Standard deviation) VPT across all participants was 3.5 V (± 1.6 V) and 3.3 V (± 1.1 V) for foot-A and B respectively. Paired samples *t* test (two tail) indicated that the difference between limbs was not statistically significant (95% CI: − 0.303/0.661, t = 0.766, df = 25, *P* = 0.451).

### SW imaging

Ultrasound SW elastography is a quantitative method for the non-invasive assessment of soft tissue biomechanics. It involves the generation of a high intensity ultrasound pulse inside the tissue that displaces a column of the tissue and generates SWs. These SWs are then tracked and their speed is measured as they propagate through different areas of the imaged tissues. At the end, SW elastography provides a 2D map of the distribution of the propagation speed of these waves in the imaging plane. In linearly elastic, homogenous and isotropic materials the measurement of SW speed enables the direct calculation of Young’s modulus (E):

1 $${\text{E }} = { 3}\uprho {\text{C}}^{{2}} ,$$

where C is the SW speed (in m/s) ρ is the tissue’s density (for soft tissues ρ ≈ ρ_water_ = 1000 kg/m^3^).

The above equation has been used in literature to estimate the Young’s modulus of soft tissues including the plantar soft tissue. However, previous work by the authors of this study demonstrated that measurements of SW speed are better suited for assessing changes and differences in stiffness rather than the measurement of the absolute values of plantar soft tissue Young’s modulus^4^. Based on that, all SW based measurements are presented as SW propagation speed (m/s) and not Young’s modulus (Pa). Readers should use Eq. (1) to compare the results presented in this study against literature where the outcome of SW elastography is presented in Pa and not in m/s.

Before imaging, the heel was cleaned using a wet wipe and its centre was marked. SW imaging was performed in the axial plane with a linear array probe (SL15-4, SuperSonic Imagine Ltd) at the centre of the heel. To ensure no compression was applied to the tissue during imaging, special care was taken to always maintain a visible layer of ultrasound gel between the probe and skin. This was necessary to avoid any misleading increase in SW speed due to externally applied compression^4,27,28^. The probe was kept in the same position for at least 10 s or until a stable SW map was achieved before capturing a frame for analysis^4–8^.

It took around five minutes to complete each imaging sequence following loading or preconditioning. During that time the participants were lying on a couch in a prone position. The potential effect of the duration of imaging on results was explored with the help of a single participant. To this end, SW speed was measured before and after ten minutes of lying still on the couch. Five images were recorded for each condition and SW speed was measured for the five circular areas of Fig. 1. Average (± standard deviation) SW speed across all five areas before and after lying on the couch for ten minutes was 4.1 m/s (± 0.2 m/s) and 4.1 m/s (± 0.1 m/s) indicating that the duration of imaging is unlikely to have influenced the results.

### SW data analysis

During data extraction, SW speed was measured in five circular areas (diameter = 5 mm). One circular area was at the centre of the loaded area, two were at the edges of the loaded area (0.5 mm left or right from the centre) and two more were outside the loaded area (1.25 cm left or right from the centre). The average SW speed within each one of these areas was automatically calculated by the ultrasound unit (AIXPLORER Ultimate MultiWave™ Ultrafast™ Imaging and ShearWave™ Elastography Ultrasound System).

During analysis, the SW speed for the unloaded tissue was calculated for each image as the average of the two circles outside the loaded area (± 1.25 cm from the centre). Similarly, the SW speed for the tissue at the edges of the loaded area was calculated by averaging the measurements for the circles at ± 0.5 cm from the centre (Fig. 1c,d). This was done to account for the increased pressure at the edges of the loaded area relative to the centre (Fig. 2a). The SW speed measurement provided by the ultrasound unit for the central circle was directly used for the tissue at the centre of the loaded area.

When the SW elastogram did not expand enough towards the borders of the region of interest to fully cover the circles outside the loaded area, a small displacement towards the centre (< 0.25 cm) was allowed to get a more reliable measurement of SW. If this displacement was not adequate to get a measurement area that is covered by the elastogram then this area was eliminated from further analysis.

Changes in SW speed were assessed by comparing baseline values against results immediately after loading and after preconditioning. In the case of foot-B the baseline values were adapted between imaging rounds to account for the possible cumulative effect of loading. More specifically, the baseline value that was used to assess the effect of loading at 80% of the reference pain threshold was the SW speed after loading at 50% and after preconditioning. Similarly, the baseline for assessing the effect of loading at the foot-specific pain threshold was the SW speed after loading at 80% and after preconditioning.

### Loading

Loading was applied at the marked centre of the heel using a handheld dynamometer (500 N, Citec) which was fitted with a standardised pain pressure applicator. The applicator had a flat circular footprint with rounded edges (diameter: 1 cm). Overloading was manually imposed with the participant lying prone on a couch by pressing the pain pressure applicator at the centre of the heel until he/she indicated the start of mild pain, at which point the heel was fully unloaded (Fig. 1b). This loading process was repeated ten times. The number of loading cycles was decided based on preliminary testing (one participant) indicating that ten cycles were likely to generate measurable stiffening without significant adverse effects.

The PFT was recorded for each cycle. The median (min. value, max. value) of the PFT for the foot tested first (foot-A) and second (foot-B) was 29 (17,110) and 34 (17,99) respectively. According to Wilcoxon signed rank test the difference between feet was statistically significant (n = 26, z = 245, *P* = 0.026).

The average PFT for foot-A was used to define the magnitude of loading for the first two (out of three rounds in total) of loading/imaging for foot-B. More specifically during the first and second round of testing the examiner loaded the marked centre of the heel of foot-B to 50% and 80% of the previously calculated PFT for foot-A. During these two rounds of testing, it was explained to the participant that loading will be applied ten times to a predefined force threshold and that loading would stop either when this threshold was reached or when they felt mild pain. Loading to the foot-specific pain threshold for foot-B was done following the same process as foot-A.

All loading and imaging processes were performed by the same examiner and were successfully completed except for one participant. SW results from foot-A of participant 18 were excluded from the analysis due to an error in loading repetitions.

### Testing/imaging sequence

The sequence of preconditioning, loading, and imaging steps are presented below in the order that they were performed during testing. The imaging steps that were used as baseline in each imaging round are also indicated.

#### Foot-A (Imaging round 1)

Imaging from step 2 was used as baseline to assess the effect of loading at the pain threshold on foot-A.

1. Preconditioning
2. SW imaging
3. Loading ten times to PFT-A
4. SW imaging
5. Preconditioning
6. SW imaging

#### Foot-B (Imaging round 1)

Imaging from step 8 was used as baseline for assessing the effect of loading at 50% of PFT-A on foot-B.

7. Preconditioning
8. SW imaging
9. Loading ten times to 50% of PFT-A
10. SW imaging
11. Preconditioning
12. SW imaging

#### Foot-B (Imaging round 2)

Imaging from step 12 was used as baseline for assessing the effect of loading at 80% of PFT-A on foot-B.

13. Loading ten times to 80% of PFT-A
14. SW imaging
15. Preconditioning
16. SW imaging

#### Foot-B (Imaging round 3)

Imaging from step 16 was used as baseline for assessing the effect of loading at the foot-specific pain threshold.

17. Loading ten times to PFT-B
18. SW imaging
19. Preconditioning
20. SW imaging

#### Foot-A (Imaging round 2)

Imaging from step 2 was used as baseline to get a sense of the longevity of overloading-induced changes in foot-A.

21. SW imaging

### Preconditioning

A preconditioning protocol of safe and clinically relevant loading was used, namely walking at self-selected speed for ≈ 80 steps. The capacity of this protocol to minimise the effect of loading history was validated by measuring SW speed after preconditioning and after the heel was exposed to different clinically relevant loading scenarios. More specifically, SW speed was measured at the left heel of a participant: (i) at baseline following initial preconditioning, (ii) after running (≈ 80 steps), (iii) after a second round of preconditioning, (iv) after performing a series of vertical jumps (24 jumps) and (v) after a final round of preconditioning. Five SW elastography images were recorded for each condition and SW speed measurements were extracted for the same circular areas as shown in Fig. 1. At the end, a single SW speed measurement was calculated for each condition by averaging measurements across all circular areas and images. To get an indicative assessment of loading magnitude, peak plantar pressure at the heel was measured for 8 walking midgait steps, 8 running midgait steps and for 10 jumps using a pressure mat (Matscan,Tekscan Inc., USA). We found that average (± standard deviation) SW speed increased from a baseline value of 4.0 m/s (± 0.1 m/s) to 4.1 m/s (± 0.1 m/s) after running only to drop back to 4.0 m/s (± 0.1 m/s) after preconditioning. It then increased again to 4.2 m/s (± 0.1 m/s) after jumping but returned to 4.0 m/s (± 0.1 m/s) after the final preconditioning round. Average peak pressure at the heel during preconditioning (walking), running and jumping was 560 kPa, 624 kPa and 440 kPa respectively. These results demonstrate the capacity of the selected preconditioning protocol to minimise the effect of loading history on plantar soft tissue SW speed.

### Statistical analysis

The normality of results was tested using the Shapiro–Wilk test. Normally distributed data are presented by their Average (± standard deviation) while non-normally distributed data by their Median (minimum value, maximum value). The statistical significance of differences was assessed using paired samples *t* test for normally distributed data and the Wilcoxon signed rank test for non-normally distributed data (a = 0.05). More specifically a number of pre-planned comparisons were conducted to test whether loading at different magnitudes can trigger significant changes in tissue stiffness that were not eliminated by preconditioning. To this end, the baseline measurement of SW speed for each imaging region was compared against the SW speed immediately after loading and after preconditioning in the same region (paired samples *t* tests, two tail). The statistical significance of differences between the limb that was tested first (foot-A) and second (foot-B) with regards to VPT and PT was also tested. All statistical analyses were conducted using IBM^®^ SPSS^®^ v26.

### Finite element modelling

A finite element analysis of the overloading scenario presented here was performed by adapting a previously published model of the healthy heel which was validated against in vivo measurements of plantar pressure^11^. The purpose of this analysis was not to produce a quantitative assessment of the absolute value of interface pressure, but to provide a relative assessment of the difference between the pressure at the edges and at the centre of the loaded area. Its results informed the decision to analyse SW speed changes at the edges of the loaded area separately to the centre and to present pain thresholds in units of force and not pressure.

The original anatomically detailed 3D model of the heel was designed based on MRI images^11^. More specifically, the left foot of a healthy participant was scanned using a 1.5 T MRI scanner and coronal T1 weighted 3D Fast Field Echo images were recorded (in-plane/out of plane resolution: 0.23 mm/1.00 mm). The 3D geometry of the heel and of calcaneus was reconstructed using ScanIP (Simpleware) and imported into Ansys (ANSYS^®^ Academic Research, Release 2021) for analysis. The final model comprised a rigid calcaneus and a bulk plantar soft tissue. The mechanical behaviour of plantar soft tissue was simulated using the Ogden hyperelastic model (1st order):

2 $$W = \frac{\mu }{\alpha }\left( {\overline{\lambda}_{1}^{ \alpha } + \overline{\lambda}_{2}^{ \alpha } + \overline{\lambda}_{3}^{ \alpha } - 3} \right) + \frac{1}{{d_{k} }}\left( {J - 1} \right)^{2} ,$$

3 $$G_{0} = \frac{1}{2}\left( {\mu {\upalpha }} \right),$$

where ${\overline{\lambda}_{1}}$, ${\overline{\lambda}_{2}}$, ${\overline{\lambda}_{3}}$ are the deviatoric principal stretches, J is the determinant of the deformation gradient and µ (Pa), α (unitless), and d_k_ (Pa^−1^) are material coefficients. Coefficient α is indirectly related to the tissue’s strain hardening/softening behaviour while both µ and α are directly linked to the material’s initial shear modulus (G_0_) through Eq. (3). Parameter d_k_ is directly linked to the material’s Poisson’s ratio (ν = 0.475). Coefficient values corresponding to a healthy plantar soft tissue were adopted (µ = 6.75 kPa, α = 12.10)^11^.

To simulate the overloading scenario presented here, the load applicator was simulated as a rigid cylinder with rounded edges. The calcaneus was rigidly fixed and an indentation force was applied to the pain pressure applicator which was equal to the average PFT across all participants and for both feet (39 N). Frictionless contact was assumed between the pain pressure applicator and the heel. At the end, the contact pressure on the surface of the heel was measured along a path on the axial plane (Fig. 3).

## Supplementary Information

Supplementary Information 1.

Supplementary Information 2.
